# Post-inflammatory Abdominal Pain in Patients with Inflammatory Bowel Disease During Remission: A Comprehensive Review

**DOI:** 10.1093/crocol/otab073

**Published:** 2021-11-08

**Authors:** Kazuya Takahashi, Iman Geelani Khwaja, Jocelyn Rachel Schreyer, David Bulmer, Madusha Peiris, Shuji Terai, Qasim Aziz

**Affiliations:** 1 Centre for Neuroscience, Surgery and Trauma, Wingate Institute of Neurogastroenterology, Blizard Institute, Barts and the London School of Medicine and Dentistry, Queen Mary University of London, London, UK; 2 Division of Gastroenterology and Hepatology, Graduate School of Medical and Dental Sciences, Niigata University, Niigata, Japan; 3 Department of Pharmacology, University of Cambridge, Cambridge, UK

**Keywords:** inflammatory bowel disease, visceral pain, brain–gut interaction, ulcerative colitis, Crohn’s disease, biologics

## Abstract

Patients with inflammatory bowel disease often experience ongoing pain even after achieving mucosal healing (i.e., post-inflammatory pain). Factors related to the brain–gut axis, such as peripheral and central sensitization, altered sympatho-vagal balance, hypothalamic–pituitary–adrenal axis activation, and psychosocial factors, play a significant role in the development of post-inflammatory pain. A comprehensive study investigating the interaction between multiple predisposing factors, including clinical psycho-physiological phenotypes, molecular mechanisms, and multi-omics data, is still needed to fully understand the complex mechanism of post-inflammatory pain. Furthermore, current treatment options are limited and new treatments consistent with the underlying pathophysiology are needed to improve clinical outcomes.

## Graphical Abstract

**Figure:**
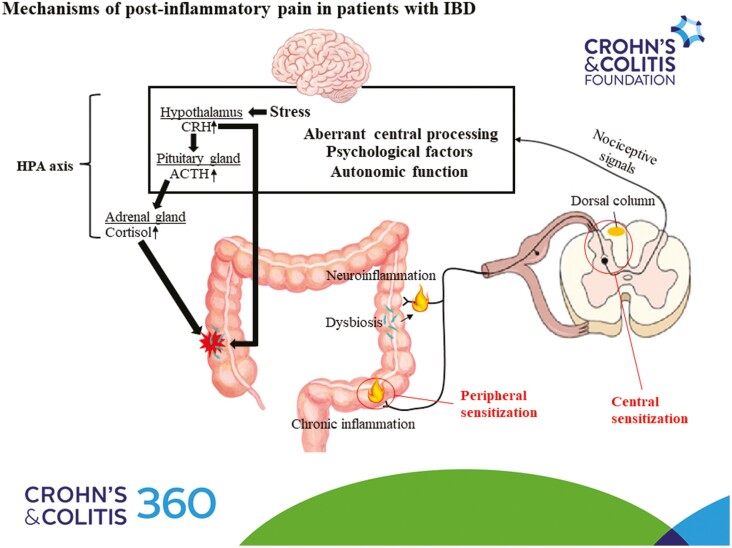

## Introduction

Inflammatory bowel disease (IBD), an umbrella term for a collection of colitides which include ulcerative colitis (UC) and Crohn’s disease (CD), is a chronic inflammatory disorder associated with remission and relapse of symptoms.^1^ Environmental factors, lifestyle, microbiota, genetic susceptibility, immune function, and psycho-physiological factors are associated with its pathogenesis.^2^ Although IBD is considered to be more common in Western countries, incidence rates of IBD continue to rise globally.^3^ As a result, there were 6.8 million cases of IBD globally in 2017.^4^

During disease flares, up to 70% of IBD patients experience abdominal pain which is associated with fatigue, depression, and reduced quality of life (QOL).^5–7^ Therapeutic goals of IBD treatment are mucosal healing in addition to clinical remission, which is referred to as deep remission.^8^ Inflammatory pain, activated by tissue damage in an acute flare of IBD, is expected to recede with a resolution of the tissue immune response^9^; however, 30–50% of IBD patients continue to experience ongoing abdominal pain even after achieving mucosal healing,^10, 11^ which is defined as post-inflammatory pain in this review. Persistent pain leads to a sustained reduction in psychological well-being and lower QOL.^6, 12^

Central and peripheral sensitization, strictures, bowel dysmotility, diet, and psychological issues have been identified as interacting risk factors.^13,14^ However, the precise mechanisms of pain during mucosal healing in IBD are complicated and not fully understood, making therapeutic approaches challenging.^11,13^ Therefore, comprehensive review of past and current knowledge can help understand the pathophysiology of post-inflammatory pain in IBD patients and provide new insights. In this narrative review, we aim to elucidate what is currently known about post-inflammatory pain in IBD to identify directions for further research and the potential for novel therapies.

## Clinical Features of IBD

Characterized by cycles of flares and quiescence, the most common clinical features of IBD include diarrhea, abdominal pain, bloody stools, and fatigue.^15,16^ UC is characterized by mucosal inflammation of the large intestine and rectum, while CD features transmural inflammation that may occur anywhere along the gastrointestinal (GI) tract.^15,17^ The most consistent feature of UC is the presence of blood and mucous mixed with stool, accompanied by lower abdominal cramping, particularly during bowel movements.^15^ In CD, intestinal luminal narrowing due to inflammation and fibrosis can result in thickening of the bowel wall, which is correlated with inflammatory activity.^17,18^ Gastrointestinal symptoms depend on the location and extent of inflammation and disease severity.^15^ Post-inflammatory pain, especially in the abdomen, persists in some patients even in periods of endoscopic remission.^13^

## Management of Pain in IBD

The National Institute for Health and Care Excellence (NICE) guidelines for chronic pain show that antidepressants improve the QOL of patients with chronic pain. Furthermore, the British Society of Gastroenterology advises that psychosocial factors such as depression/anxiety, stress, and sleep disturbance may be addressed through psychological interventions such as cognitive behavioral therapy, which may improve QOL after treating the underlying IBD and identifying obvious physiological causes of pain.^19, 20^ However, to the best of our knowledge, there are no guidelines specific to the management of post-inflammatory abdominal pain in IBD.

Acetaminophen (37%), Nonsteroidal Anti-inflammatory Drugs (NSAIDs) (13%), and opioids (16%) are mainly used for the treatment of pain in IBD.^5^ However, these medicines typically have limited efficacy against visceral pain, which is consistent with the NICE guidelines. Importantly, the side effects associated with these treatments can exacerbate symptoms and reduce patient compliance. For example, NSAIDs can cause serious side effects such as GI ulcers and bleeding, causing abdominal pain and anemia,^21–23^ and are linked to exacerbation of IBD.^9,22–24^ Similarly, opioids cause gastrointestinal side effects, particularly nausea, vomiting, and constipation, in addition to having an addictive quality which may lead to misuse and potentially illegal drug use.^25–27^ Furthermore, extended opioid use among patients with IBD is associated with higher total health care costs.^28^

Thus, the current pharmacological treatment options for post-inflammatory abdominal pain are limited, and some of them can exacerbate symptoms and disease activity if misused. The lack of effective pain management strategies coupled with the complex and multifactorial nature of post-inflammatory pain in IBD makes treatment more difficult.^11^ Therefore, a robust treatment guideline and new effective treatments are warranted.

## Neuroanatomy of the Brain–Gut Axis

Post-inflammatory abdominal pain in IBD involves a complex and multifaceted interplay of overlapping factors associated with the brain–gut axis.^24^ Understanding the basic neuroanatomy of the brain–gut axis will help consider the mechanisms of post-inflammatory abdominal pain. Therefore, we first summarize the normal neuroanatomy of the extrinsic sensory nerves, which play a central role in nociception in the digestive tract.

The digestive tract receives innervation by extrinsic sensory nerve endings that project to the central nervous system (CNS) via vagal and spinal nerves, respectively.^29^ The endings of these nerves can be seen in all layers of the gut wall. Sensory nerves, referred to as spinal afferents, which project to the dorsal horn of the spinal cord via cell bodies located in the dorsal root ganglia (DRG) function largely as polymodal nociceptors that are responsive to noxious chemical and mechanical stimuli. This includes the presence of a silent nociceptor population, which only display mechanosensitivity following the application of “priming” inflammatory mediators such as prostaglandins, yet play a major role in nociception.^29^ Vagal afferents, although also polymodal in nature, respond to non-noxious levels of mechanical stimuli with saturated stimulus response function across the noxious range (>30 mmHg). Although also responsive to noxious chemical stimuli such as capsaicin and protons, vagal afferents are largely thought to serve more physiological sensory functions such as the initiation of vago-vagal reflexes and the sensation of satiety due to the sensitivity of vagal afferents to gut hormones such as cholecystokinin (CCK) and peptide YY (PYY). (Please refer to the later section regarding the function of vagal afferent nerves.)

Although CD can be found throughout the gut, it is most frequently found within the ileocecal and colonic regions. Furthermore, UC exclusively affects the colon and rectum, the innervation of which warrants a particular focus. Spinal afferent fibers can be further characterized into splanchnic and pelvic afferents (based on the nerve trunks in which they project). The splanchnic nerve innervates the proximal to the distal colon while the pelvic nerve innervates only the rectum.^30,31^ The cell bodies of splanchnic and pelvic afferents are found in the DRG of thoracolumbar (T10-L1) and lumbosacral (L6-S1), respectively.^30^ The vagal nerve also innervates the proximal to the transverse colon.

Splanchnic and pelvic afferents are pseudo-unipolar neurons,^30^ whose central axons predominantly terminate in superficial (lamina I) and deeper (lamina V and X) layers of the dorsal horn of the thoracic, lumbar, and sacral spinal cord, forming synapses with second-order neurons.^30,32^ Visceral information ascends via the spinoreticular, spinomesencephalic (which includes the spinoparabrachial tract), spinohypothalamic, and spinothalamic tract.^31,33^ Among these tracts, the spinothalamic tract is related to conscious sensation, which then projects to the thalamus and higher cortical areas such as the somatosensory cortices, the anterior cingulate cortex (ACC), and the insula.^31^

The periaqueductal gray (PAG) in the midbrain is critical for the descending pain modulation system, providing the site of origin for neuronal pathways which project to the spinal dorsal horn via relays within the rostral ventromedial medulla (RVM).^34^ This system receives direct nociceptive information from the parabrachial nuclei and the spinoreticular pathway.^34,35^ Moreover, the prefrontal executive control areas (medial and dorsolateral prefrontal cortex), anterior medial cingulate cortex, and emotional arousal areas (anterior insula, subgenual ACC, and amygdala) have a strong connection with autonomic and endocrine response structures such as the hypothalamus and brain stem nucleus such as nucleus tractus solitarius (NTS). These are involved in the top-down modulation of descending pain pathways.^34^ The sensitivity of spinal dorsal horn neurons is modulated by the activation of PAG, RVM, or top-down modulation.^34,35^ Thus, descending pain modulation mechanisms participate in mediating visceral pain perception via the brain–gut axis.

## Pathophysiologic Factors

A number of pathophysiological factors related to the brain–gut axis that may have an impact on the development of chronic pain in IBD have been identified and described below.

### Peripheral and Central Sensitization in IBD

**Figure 1.**
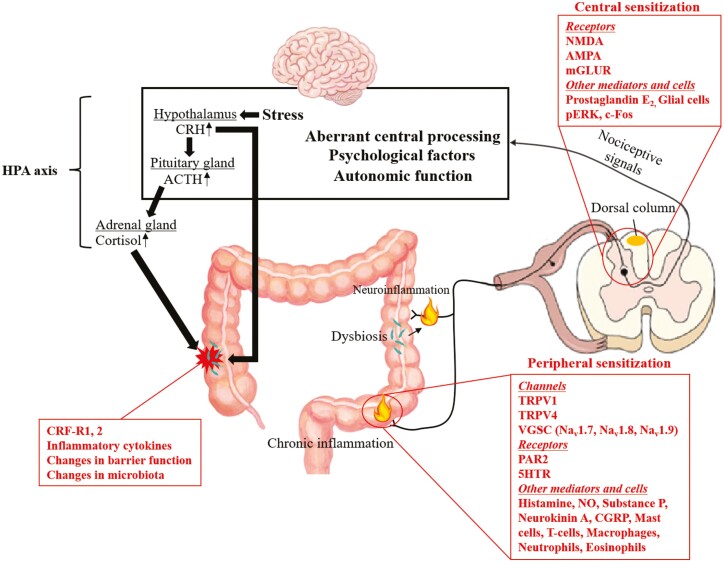
Schematic summary of suggested mechanisms of post-inflammatory abdominal pain in IBD. (Reproduced with permission from Aziz. Pain 2009^29^). Abbreviations: 5-HT, 5-hydroxytryptamine; ACTH, adrenocorticotropic hormone; AMPA, α-amino-3-hydroxy-5-methyl-4-isoxazole propionic acid; CGRP, calcitonin gene-related peptide; CRF-R, corticotrophin-releasing factor receptor; CRH, corticotropin-releasing hormone; HPA axis, hypothalamic–pituitary–adrenal axis; mGLUR, metabotropic glutamate; NMDA, N-methyl-D-aspartic acid; NO, Nitric oxide; PAR2, protease-activated receptors 2; pERK, phosphorylated extracellular signal-regulated kinase; TRPV1, transient receptor potential vanilloid 1; TRPV4, transient receptor potential vanilloid 4; VGSC, voltage-gated sodium channels.

Visceral hypersensitivity, which is often evaluated by measuring rectal sensitivity to mechanical, thermal, or electrical stimuli, is thought to be one of the mechanisms of abdominal pain in patients with IBS.^36^ In contrast, our recent meta-analysis suggested that visceral hypersensitivity is not a universal finding in patients with quiescent IBD except during active flares of the disease.^37^ However, considering the high concomitant rate of IBS among IBD patients, visceral hypersensitivity is a likely cause of post-inflammatory abdominal pain.^38–41^ Visceral hypersensitivity is caused by sensitization or alteration of the peripheral and central pathways during the IBD process.^42^ We here describe the possible mechanisms and associated risk factors of post-inflammatory visceral hypersensitivity pain in IBD. The pain pathways discussed in this review are summarized in Figure 1.

#### Molecular pathways of peripheral sensitization

To date, there have been considerable numbers of reports showing the underlying molecular mechanisms of peripheral sensitization in animal models of colitis and patients with IBD.^38–41^

#### Transient receptor potential vanilloid 1

Transient receptor potential vanilloid 1 (TRPV1) is a noxious heat-sensitive ion channel gated by other painful chemical stimuli such as capsaicin, protons, and heat (>43°C)^39,43–45^ that is widely expressed in visceral afferent neurons and implicated in the presence of abdominal pain in GI disorders.^44^ For example, TRPV1-immunoreactive nerve fibers in biopsy specimens from the colon of IBS patients were on average 3.5 times more frequent than in control subjects.^46^ In addition to the transduction and relay of noxious stimuli, the activation of TRPV1 triggers the release of neuropeptides such as substance P and calcitonin gene-related peptide (CGRP) from the peripheral nerve terminal of sensory neurons, leading to neurogenic inflammation in the colon.^39,40,47,48^ Moreover, pro-inflammatory mast cell mediators such as histamine mediate sensitization of TRPV1^31,39,49,50^ and TRPV 1 expression is increased in animal models of inflammation-related hypersensitivity.^51,52^ Furthermore, mucosal TRPV1 expression is positively correlated with abdominal pain severity of quiescent IBD patients (with IBS-like symptoms).^48^ Taken together, TRPV1 would be a key modulator in post-inflammatory abdominal pain in IBD.

#### Transient receptor potential vanilloid 4

Transient receptor potential vanilloid 4 (TRPV4) is a mechano-transductive osmo-sensitive cation channel that is expressed in sensory neurons and activated by a wide range of stimuli including stress, non-noxious warm temperatures, acidity, phorbol esters, and downstream metabolites of arachidonic acid.^53–55^ TRPV4 has been implicated in the pathophysiology of inflammation and visceral hypersensitivity in patients with IBD and IBS.^56,57^ Activation of TRPV4 in the GI tract has been linked to increased epithelial barrier permeability, chemokine release, and recruitment of immune cells.^56,58^ In the GI tract, TRPV4 is found expressed in ileal and colonic sensory nerve fibers, DRG neurons, and fine nerve fibers associated with blood vessels in the submucosa and serosa.^30,31^ Research suggests that TRPV4 mRNA is expressed up to three times more in colonic sensory neurons compared to other visceral and somatic sensory neurons.^54^ TRPV4 mRNA expression and TRPV4 immunoreactivity in human colon biopsies are significantly increased in IBD patients compared to healthy controls.^54,58,59^ In mouse models of experimentally induced colitis, administration of TRPV4-selective and non-selective antagonists significantly reduce macroscopic damage and myeloperoxidase activity (a pro-inflammatory marker) as well as pain.^59^ The administration of TRPV4 agonists shows the opposite effect with marked increases in measures of colonic inflammation in treated versus. untreated mice.^60,61^ Furthermore, intestinal inflammation is decreased in TRPV4 knockout (KO) mice compared to wild type (WT) mice in a dextran sulfate sodium (DSS) colitis model.^60^ Thus, there is evidence that activation of TRPV4 has an inflammatory and pro-hypersensitivity function during colitis, and the evidence that TRPV4 antagonist had a protective effect on animal models of colitis makes it a promising therapeutic target.^62^

#### Nitric oxide pathway

Nitric oxide (NO) is a signaling molecule, which is an important regulator of gastrointestinal integrity, host immune response, and tissue repair. There are three isoforms of nitric oxide synthase (NOS): endothelial (eNOS), neuronal (nNOS), and inducible (iNOS).^53,63^ These isoforms are often co-expressed in multiple cell types and all use arginine as the substrate for NO synthesis.^63^ Arginosuccinate lyase (ASL) is the only enzyme in the human body that is able to endogenously generate arginine.^64^ As such, loss of ASL leads to metabolic restriction of arginine for all NOS-derived NO.^65^ In ASL KO mice with DSS colitis on a restricted arginine diet, inflammation and colitis severity are significantly increased compared to WT mice.^66^ In another study with a colitis mouse model, cell-autonomous production of NO by enterocytes alleviates colitis as part of the innate immune response.^66^ Furthermore, some implicate increased NO levels in IBD patients with increased levels of gastric inflammation.^67–69^ Therefore, NO pathway is considered to play a role in inflammation in IBD patients.

Although the results are conflicting, several studies have demonstrated the involvement of NO pathways in visceral hypersensitivity in human and animal models. In rat models, antinociceptive effect of NO pathways on visceral hypersensitivity has been shown.^70,71^ For example, Shamshiri et al. demonstrated that lithium inhibited visceral hypersensitivity in a rat model with acetic acid-induced colitis and that the effect was lowered by NOS inhibitors, suggesting the antinociceptive effect of NO pathway in visceral hypersensitivity.^72^ In contrast, in a randomized controlled study, NOS inhibitor increased the threshold for discomfort/pain in IBS patients.^73^ This conflicting data may derive from differences between subjects. However, it is likely that the NO pathway plays a role in the pathophysiology of visceral hypersensitivity.

#### 5-Hydroxytryptamine receptors

The 5-hydroxytryptamine (5-HT) receptors are comprised of seven subfamilies (5-HT_1-7_) and fourteen receptor subtypes.^74^ The exact role of each receptor subtype is unknown, but it is clear that serotonin acts as a neuromodulator with both nociceptive and antinociceptive effects.^75^ This variance is dependent on the cell type, receptor subtype, and anatomical region where the action takes place.^76^ In the GI tract, 5-HT is produced and stored in the mucosal entero-chromaffin (EC) cells and secreted in response to luminal stimuli.^77^ Persistent increases in 5-HT within the intestinal mucosa can facilitate spinal afferent mediated visceral nociceptive processing via activation of 5-HT type 3 receptors (5-HT_3_R) in animal models. Activation of 5-HT_3_R releases substance P, neurokinin A, and CGRP from primary afferents thus contributing to the pathogenesis of visceral hyperalgesia.^78–80^ In contrast, acute exposure to 5-HT is proposed to have an antinociceptive effect by activating vagal 5-HT_3_R leading to modulation of the pain response in the colon of a rat model.^81^

Several animal studies have demonstrated a link between colonic inflammation, the visceral pain response, and increases in 5-HT_3_R expression.^78,82,83^ In a rat model of visceral pain, longer 5-HT exposure leads to increased visceromotor response to colorectal distension in a time-dependent manner.^81^ Interestingly, a recent study in rats suggested visceral hypersensitivity due to increased 5-HT signaling and 5-HT_3_R activation was female sex-specific.^84^

Changes in the number of EC cells and the levels of 5-HT in the serum samples from IBD patients have previously been documented.^85,86^ Colonic biopsy specimens from IBD patients also demonstrate an upregulation of 5-HTR_3_ and, more recently 5-HTR_7_ expression with associated increases in intestinal inflammation.^87,88^ Whilst this demonstrates a role for serotonin signaling in IBD pathogenesis, it remains to be seen if this also correlates with the development of chronic pain in these patients. However, treatment with 5-HT_3_R antagonists has shown to provide symptomatic relief of abdominal pain in patients with IBS.^89^

#### Voltage-gated sodium channel

Voltage-gated sodium channels (VGSCs) are found throughout nerves in the body and are involved in the initiation and propagation of action potentials in DRG responsible for pain transmission.^53,90^ The channels Na_v_1.7, Na_v_1.8, and Na_v_1.9 are exclusively located in the PNS and these isoforms play a major role in the modulation and development of GI pain and peripheral sensitization (comprehensively reviewed by Coates et al.).^90^ There are several human cohort studies that have demonstrated differences in pain perception between participants who have naturally occurring mutations in the genes encoding Na_v_1.7, Na_v_1.8, and Na_v_1.9 and normal controls.^91–94^ Furthermore, colonic afferent activation by supernatants derived from inflamed human tissue was blocked in Na_v_1.9 KO mice, implying the significance of Na_v_1.9 in visceral nociception.^95^ Moreover, colonic afferent activation by supernatants derived from inflamed human tissue was greatly reduced in Na_v_1.9^−/−^ mice.^95^ These results demonstrate that Na_v_1.9 is required for the persistence of responses to intense mechanical stimulation, contributes to inflammatory mechanical hypersensitivity, and is essential for activation by noxious inflammatory mediators, including those from diseased human bowel. These observations indicate that Na_v_1.9 represents a high-value target for the development of visceral analgesics. Furthermore, in a study targeting patients with IBD, a link between a genetic variant of SCN10A, a gene coding Na_v_1.8, and abdominal pain perception was found.^96^ These previous studies demonstrate that targeting specific VSCGs could be a pathway for improving the diagnostic and therapeutic management of chronic abdominal pain in IBD patients.

#### Central sensitization pathways

Central sensitization is defined as increased responsiveness of nociceptive neurons within the CNS to their normal or sub-threshold afferent input.^97^ Central sensitization can lead to pain hypersensitivity which manifests in dynamic tactile allodynia, secondary punctate or pressure hyperalgesia, after-sensations, and enhanced temporal summation.^98^ Models of abdominal pain in IBD suggest that sensitization occurs as a result of persistent intestinal inflammation. Peripheral sensitization is considered to be the main driver of pain in acute flares in IBD whilst central hypersensitivity is the key driver in post-inflammatory chronic pain experience.^9,11^

The existence of referred pain in human and animal models of colitis provides strong evidence for CNS involvement in the development of abnormal pain. Referred pain is to likely occur when there is overlap between visceral and somatic pathways within the CNS, which is common within the spinal dorsal column.^11^ Recurrent visceral stimulation by extrinsic afferent neurons activates intracellular signaling pathways within the spinal dorsal horn neurons. Exaggerated responses to innocuous and noxious inputs occur when there is an upregulation of excitatory synaptic responses and a downregulation of descending inhibitory responses.^99^ Under inflammatory conditions such as colitis, ion channels that are responsible for the mechano-transduction and generation of action potentials related to pain are upregulated.

The main excitatory neurotransmitter is glutamate. Glutamate activates the N-methyl-D-aspartate (NMDA) receptors, kainate, α-amino-3-hydroxy-5-methyl-4-isoxazole propionic acid (AMPA) receptors, and metabotropic glutamate (mGLUR) receptors expressed by the dorsal horn neurons.^99^ Hyperstimulation of NMDA receptors by glutamate leads to their phosphorylation or transcriptional upregulation. This can result in long-lasting neuronal excitability in the absence of inflammation.^9,100^ Another important mediator of central sensitization is prostaglandin E_2_ (PGE_2_) and the PGE_2_ receptor. PGE_2_ suppresses glycinergic transmission via activation of PGE_2_ receptor which results in the release of dorsal horn nociceptive neurons from inhibition by glycinergic neurons. This then facilitates nociceptive input to the spinal cord.^99^

Glial cells have been implicated in the development of central sensitization.^101^ Microglia are activated in the spinal dorsal horn, especially in superficial lamina I-II, where nociceptive input is mainly processed in response to injury or inflammation.^102,103^ In animal models of neuropathic pain, spinal astrocytes become activated following inflammation. Inhibiting the activation of astrocytes correlates with a decrease in inflammatory hyperalgesia. Active glial cells produce several pro-inflammatory mediators such as TNF-α which contribute to the sensitization of nerve fibers in the dorsal horn.^103^

There is currently no direct method to measure central sensitization in humans. However, there are a large number of indirect measures that are used clinically. These include questionnaires (for example, Central Sensitization Index (CSI) and Pain Sensitivity Questionnaire (PSQ)), bedside sensory testing (hypo- or hyper-phenomena, wind-up like pain, and after-sensation), and mapping of areas with sensory abnormalities. In an experimental setting, rectal stimulation by balloon distension is often used to evaluate central sensitization. Chemical or thermal stimulation are also used but are not as common as balloon distension.^36,104^

#### Aberrant central processing of visceral pain

Chronic pain is known to cause structural and functional changes in the brain. Imaging studies have previously noted hyper-excitability in pain-associated areas which include the limbic system, cingulate gyrus, the somatosensory cortex, and the insula.^105^ In patients with quiescent UC, the lateral frontal region and the dorsal pons/PAG have shown activation by PET.^106^ One recent study in rats proposed that persistent colonic inflammation results in functional coupling between the ACC and the central medial thalamic nucleus and this coupling could play a role in the pathophysiology of visceral hyperalgesia in IBD.^107^ On a molecular level, two markers of neural inflammation c-Fos and phosphorylated extracellular signal-regulated kinase (pERK) are commonly used to evaluate CNS involvement in pain processing in animal models. These markers are found elevated in the PAG, dorsal raphe nucleus, pontine parabrachial nucleus, locus coeruleus, and nucleus of the solitary tract following colonic inflammation.^108^

Transcranial direct current stimulation (tDCS) has been shown to reduce pain in IBD patients with chronic abdominal pain.^109^ Following treatment with tDCS, a small cohort of patients with IBD and chronic abdominal pain showed higher functional connectivity in the visual medial and right frontoparietal network.^110^ Greater connectivity in these areas is associated with improved pain modulation and this, taken with the changes observed following tDCS, suggests that these areas may be associated with chronic abdominal pain processing.^110^

### Autonomic Nervous System

The autonomic nervous system (ANS), which consists of the sympathetic nervous system (SNS) and PNS, is also associated with pain processing regulation.^59,60^ SNS and PNS are considered pro- and antinociceptive, respectively.^111^ The imbalance between the sympathetic and parasympathetic arms (i.e., the decreased activity of parasympathetic tone) often occurs in pain disorders.^112–114^ Patients with IBD tend to show lower PNS activity than healthy subjects,^115^ and such dysfunction of ANS is also suggested to be associated with post-inflammatory abdominal pain in IBD.^116^

The vagus nerve is the main branch of the PNS and innervates from the esophagus to the proximal colon. The efferent vagal nerve arises in the dorsal motor nucleus (DMNx) in the medulla.^117^ On the other hand, vagal afferent fibers commence from the digestive tracts’ mucosal or muscle layers and have cell bodies in the nodose ganglia. Although visceral pain arises exclusively from spinal afferents and vagal afferent fibers are considered to have a much lesser role in nociception, they still work as a sensor of primarily physiologic non-noxious stimuli such as satiety, nausea, and fullness, convey interoceptive information, and regulate autonomic function.^29,118,119^ The role of vagal afferents in chemo- and mechano-sensation is mediated by luminal stimuli including mechanical distention, chemical stimulation, and intestinal microbiota with their metabolites.^120^ Vagal afferents relay sensory information to NTS in the medulla, which is often referred to as the “dorsal vagal complex” together with DMNx, an area of key importance in autonomic and limbic responses to autonomic sensory input.^115^ From the dorsal vagal complex, visceral information ascends to subcortical areas (i.e., the hypothalamus, thalamus, and amygdala via the parabrachial nucleus) and then is relayed to higher cortical areas (i.e., the insula cortex, cingulate, and prefrontal cortices).^115^ These brain areas are referred to as the central autonomic network (CAN),^121^ which subsequently can modulate visceral function by descending neural command and plays a significant role in bi-directional effect between the CNS and the peripheral digestive tract.^122,123^

Brain imaging studies in migraine patients demonstrated that activation of PNS increases the NTS activity and increases connectivity to the anterior insula and ACC, which are key areas for pain processing and descending analgesia.^124,125^ Thus, PNS is thought to exert an antinociceptive effect, possibly due to vagal nerve-mediated activation of key brain areas implicated in descending pain modulation pathway^126,127^ (refer to “Neuroanatomy of the Brain–Gut Axis” section). This pathway will be a potential target of post-inflammatory pain treatment.

### Stress and the Hypothalamic–Pituitary–Adrenal (HPA) Axis

Stress, defined as an organism’s response to a change in its homeostatic environment, is also an important factor in the complex mechanisms of brain–gut interaction. Psychological stress activates the HPA axis, causing the release of corticotrophin-releasing factor (CRF) from the paraventricular nucleus of the hypothalamus.^128,129^ CRF then promotes the release of adrenocorticotropic hormone from the pituitary into the systemic circulation, which causes the synthesis and release of cortisol from the adrenal cortex.^128^ CRF is a 41 amino acid peptide and acts through binding to receptor subtype 1 (CRF-R1) and CRF receptor subtype 2 (CRF-R2).^130–132^ CRF-R1 and CRF-R2 are localized in the enteric nervous system, EC cells, and immune cells such as mast cells, eosinophils, and T-helper lymphocytes in the brain and peripheral organs including GI tracts.^131,133^ CRF mediates mast cell degranulation in the colon in experimental animals and humans through CRF-R1, which then leads to the release of mediators such as histamine, 5-HT, tryptase, prostaglandin E2, and nerve growth factor.^133–135^ These mediators alter intestinal barrier function, resulting in increased permeability of the GI tract, and activate spinal sensory afferents, which subsequently leads to visceral hypersensitivity.^135–137^ On the other hand, CRF-R2 is thought to have an inhibitory effect on CRF-R1 activation by regulating neurotransmitter release by myenteric neurons.^132,138^

Although the role of cortisol on visceral pain remains not fully understood, cortisol is thought to activate resident immune cells and extrinsic primary afferents within the GI tract to promote peripheral sensitization.^139^ A recent randomized control study showed that administration of oral hydrocortisone enhanced visceral pain sensitivity in healthy volunteers.^140^

### Microbiota

Signaling molecules from gut microbiota, including by-products of microbiota, metabolites, neurotransmitters, and neuromodulators, may play an important role in mediating neuroinflammation, which could result in peripheral and central sensitization.^141^ There are numerous preclinical and clinical reports about the effect of microbiota on visceral pain. For example, the antibiotic treatment decreased visceral pain induced by intraperitoneal acetic acid or intracolonic capsaicin in a mouse model.^142^ Furthermore, administration of *Lactobacillus* strains induced the expression of µ-opioid and cannabinoid receptors in intestinal epithelial cells and mediated analgesic functions in the GI tract in a mouse model.^143^ Clinical studies also demonstrate probiotics, such as *Lactobacillus rhamnosus* GG, a mixture of *Bifidobacterium infantis* M-63, *breve* M-16V, and *longum* BB536, and *L.acidophilus* NCFM, were effective on functional abdominal pain.^141,144–146^ Notably, fecal microbiota transplantation from IBS patients with visceral hypersensitivity to germ-free rats caused visceral hypersensitivity in the rats.^147^ All these previous studies indicate the association between gut microbiota and visceral pain. Given that the disease pathogenesis of IBD shows a strong association with changes in gut microbiota,^148^ changes in gut microbiota are likely involved in post-inflammatory pain in IBD patients.

### Psychological and Psychosocial Factors

Post-inflammatory pain due to IBD significantly impacts patients’ health-related quality of life (HRQOL).^5,6^ A number of psychological factors are associated with pain in adult and pediatric patients including depression, anxiety, and pain catastrophising.^21,149,150^ Several studies demonstrate that a higher percentage of patients with IBD suffer from anxiety and depression than those of the general population.^21,151,152^ A study of hospital-based patients showed that IBD patients suffering from chronic pain were more likely to have lower psychological well-being demonstrated by increased anxiety and depression scores.^12^ Furthermore, patients with UC in remission that suffer from IBS-like symptoms (including abdominal pain) demonstrate higher levels of anxiety, depression, and stress than those patients in remission without IBS-like symptoms.^153^

Positive psychological factors (self-efficacy and psychological well-being) are associated with less pain; these positive factors may improve resilience and therefore ability to cope with pain.^154^ In contrast, maladaptive coping strategies such as self-blame, denial, withdrawal behavior have been correlated to increased severity of pain.^21^

Pain elicits an emotional response in the individual and recurrent episodes have been hypothesized to result in hypervigilance.^9^ Hypervigilance heightened by psychological stress has been shown to lead to the disinhibition of the descending pain modulation pathways resulting in increased pain levels even in the absence of inflammation.^9^

Pain due to IBD can combine with a number of social factors such as employment status, social deprivation, social attachment style, and early childhood adversity to decrease a patient’s HRQOL.^155,156^ One study of Korean patients with inactive IBD found that pain was associated with socioeconomic deprivation.^157^ Although strong associations exist between psychosocial factors and chronic pain, it is unclear whether this relationship is reciprocal as opposed to causal.^9,154^

## Is There a Role for Genetics in Post-Inflammatory Pain?

Chronic pain is thought to have a significant genetic component. For example, chronic pain due to IBS has been shown to have a heritable aspect of around 25% in twin studies.^158,159^ Several genome-wide-association studies (GWAS) which investigate genetic variants of widespread chronic pain in large general population cohorts have been conducted.^123,160^ However, it is currently unclear whether the mechanisms underlying chronic pain are common or different between different body sites.^161^ One recent GWAS of multisite chronic pain (MCP) identified 76-independent SNPs associated with MCP. MCP showed a positive genetic correlation with autoimmune disorders such as rheumatoid arthritis but did not show any association with IBD.^160^ Interestingly, another GWAS was able to demonstrate a genetic correlation between neuroticism and stomach/abdominal pain although this study did not correlate these findings to specific diseases such as IBD.^161^

Recently, Swiss IBD Cohort group has shown that 2 SNPs rs1042713 (located on the ADRB2 gene) and rs4663866 (close to the HES6 gene), which are significantly associated with IBS, were also associated with abdominal pain level in patients with UC.^162^ This result may explain the high prevalence rate of IBS among IBD patients. Furthermore, three candidate genes - transient receptor potential vanilloid type 3 (*TRPV*3), prostaglandin-endoperoxide synthase isoenzyme 2 (*PTGS2),* and mitogen-activated protein kinases (*MAPK14*) have been associated with increased pain burden in newly diagnosed pediatric IBD patients.^163^*TRPV3* belongs to the same family as *TRPV1* which has been implicated in visceral hypersensitivity of the GI tract.^39^

## Have Any Endophenotypes Been Identified and do They Predict Outcomes in Terms of Disease, Severity, Pain Prevalence, and Treatment Responses?

Endophenotypes are objectively measurable components that are not easily observed on the surface.^164,165^ Ideally, these components should be present even when an individual is not displaying symptoms and should show heritability.^164^ Evidence suggests that there are two reproducible pain clusters in humans, that is, pain cluster 1 (PC1) and pain cluster 2 (PC2).^166^ Individuals in PC1 can be characterized by high neuroticism, anxiety, serum cortisol and SNS tone, low PNS tone, and an overrepresentation of the S allele of serotonin transporter length polymorphism (5-HTTLPR). The S allele of 5-HTTLPR is associated with lower 5-HTT expression and consequently anxiety and low mood in healthy individuals.^167^ This is in contrast to individuals in PC2 who show the opposite profile. Healthy individuals clustered in PC1 demonstrate a lower threshold to acute pain when challenged than those individuals in PC2.^166^ PC1 has greater representation in patients with functional chest pain versus PC2,^168^ but this has not been investigated in individuals with IBD and therefore further studies are required to determine how these endophenotypes would influence disease severity, pain prevalence, and treatment response in IBD patients.^169^

## Do Biologics Influence the Prevalence of Post-inflammatory Abdominal Pain?

Recently, biologics targeting inflammatory mediators have improved the disease management of IBD, allowing more mucosal healing and reducing surgery rates in patients with moderate to severe IBD.^170,171^ Anti-tumor necrosis factor-alpha (TNF-α) agents (infliximab, adalimumab), interleukin 12 and 23 antibody (ustekinumab), and an α4β7 integrin antibody (vedolizumab) are currently available both for CD and UC in the United Kingdom. Furthermore, another anti-TNF-α agent (golimumab) and selective Janus kinase inhibitor (tofacitinib) can also be used for UC.

There are enormous numbers of clinical trials of those biologics for IBD. Although disease activity (e.g., clinical remission rate, mucosal healing rate, etc.) is often the primary outcome in those clinical trials, patients’ reported outcomes including abdominal pain have also been assessed by instruments related to HRQOL, such as Inflammatory Bowel Disease Questionnaire (IBDQ), 36-Item Short-Form Health Survey (SF-36), or EQ-5D questionnaire.^13,172–178^ For example, in phase 3, randomized, double-blind, placebo-controlled trial of vedolizumab for UC (GEMINI 1), greater proportions (6.9-19.9%) of vedolizumab-treated patients had clinically significant improvement for all those instruments compared to placebo.^172,174^ The improvement of patients’ reported outcomes related to their HRQOL is also seen in other studies.^174,176, 177^ Furthermore, in a post hoc analysis of 3 clinical trials (GEMINI 1, 2, and 3), vedolizumab reduced abdominal pain subscore in CDAI by 32.8% from the baseline in patients with CD who were TNF antagonist-naïve.^179^

Thus, the results of previous studies imply that biologics are effective for acute inflammatory abdominal pain in patients with IBD. Also, results from the other studies showing the effectiveness of biologics on extraintestinal pain support the potential of biologics for the treatment of abdominal pain in IBD.^180,181^ However, questionnaires used in those clinical trials are comprehensive and not pain-specific. Therefore, the evaluation of pain (e.g., quantitative assessment, location of pain, duration of pain, etc.) has not been detailed enough in biologics trials. Furthermore, biologics are usually introduced to patients with moderate to severe IBD, and the clinical trials of biologics have targeted such patients. Therefore, although biologics may improve abdominal pain due to acute inflammation, the effect of biologics on post-inflammatory abdominal pain and its prevalence remains to be understood.

Despite a paucity of clinical evidence so far, the results of some preclinical and clinical studies have implied that TNF-α targeting therapy, which is thought to be an important mediator in inflammatory and neuropathic pain,^182, 183^ might have an analgesic effect on post-inflammatory abdominal pain. Vivinus et al. demonstrated that IBS-like symptoms in patients with quiescent IBD are associated with epithelial barrier disruption and that persistently increased expression of TNF-α in colonic mucosa might contribute to this disruption.^184^ Furthermore, Hughes P.A. et al. reported that pro-inflammatory cytokines, including TNF-α, from diarrhea-predominant IBS patients caused mechanical hypersensitivity in the colon in the murine model.^185^ Intriguingly, mechanical hypersensitivity was reduced by infliximab in their study.^185^ Since IBS is a common comorbidity of IBD,^186^ the result of this study implies the potential effects of infliximab on abdominal pain in patients with IBD. TNF-α is also reported to sensitize TRPV1 in the spinal cord and trigeminal neurons.^183, 187, 188^ Although the precise interaction between TNF-α and TRPV1 expressed in the visceral neurons is not fully understood, TNF-α targeting therapy may also change pain perception via TRPV1.

## Exploration of New Treatments for Post-inflammatory Abdominal Pain

As discussed above, peripheral and central sensitization and various psycho-physiological factors are associated with post-inflammatory abdominal pain. The potential of new pharmacological and non-pharmacological treatments targeting those factors has been explored.

### Pharmacological Treatment Targeting TRP Channel Family

To date, several TRPV1 antagonist compounds have been tested in animals and humans.^31^ However, first-generation polymodal TRPV1 antagonist caused marked hyperthermia, terminating clinical development, and therefore, did not progress clinically.^189–192^ Second-generation thermoneutral TRPV1 antagonists that selectively block channel activation by capsaicin do not cause thermal side effects, however, the analgesic effect was limited.^193^ Thus, the possible utility of TRPV1 antagonists for the treatment of pain in IBD patients is still far from clear.

An alternative approach may be the use of TRPV1 agonists such as capsaicin to desensitize sensory nerves and this approach has shown efficacy in GI diseases such as IBS or functional dyspepsia (FD).^31,194,195^ In a comparable manner, cross-desensitization therapy by menthol is another potential treatment for abdominal pain, which is reported to have an analgesic effect in patients with IBS.^31,196^ Menthol is known to be an agonist of the transient receptor potential melastatin 8 (TRPM8), another member of the TRP channel family. Activation of TRPM8 inhibits chemo- and mechanosensory responses of TRPV1 and subsequently exerts an analgesic effect, which is referred to as cross-desensitization.^31^ Thus, desensitization and cross-desensitization therapies may treat visceral pain.

More recently, the potential of treatment targeting associated mediators of TRPV1 has been explored.^31,49,50^ For example, Perna E. et al. reported that resolvins, which are endogenous inhibitors of TRP channels that suppress thermal and mechanical hypersensitivity, restored pain responses to colorectal distension in preclinical models of visceral hypersensitivity in a post-inflammatory rat model.^50^ They also showed that resolvins normalized TRPV1 activation of submucosal neurons in rectal biopsies of patients with IBS.^50^ Somatostatin, which counteracts pro-inflammatory neuropeptides such as CGRP and substance P, is another candidate for a treatment drug.^31,197^ Somatostatin interacts with 5 G-protein-coupled receptors (sst1–5), and selective agonists to sst1 and ss2 improved visceral hypersensitivity in the mice model.^197^ These new compounds seem to have promising potential thus far.

### Non-Pharmacological Treatment – Psychological Interventions

As discussed in another section, psychological factors such as stress, depression, anxiety, coping skills, and catastrophizing are risk factors for post-inflammatory abdominal pain in patients with IBD.^21^ From these perspectives, various non-pharmacological treatment approaches have been tested. Gracia-Vega et al. reported the favorable effect of stress-management training on abdominal pain in a randomized controlled study with 45 patients with CD in remission.^198^ The effectiveness of other psychological interventions such as training for coping skills, cognitive behavioral therapy, and relaxation training are also reported.^199–201^ The current limitation is that the qualities of those studies were low to medium due to the limited sample size or study design (i.e., disease activities of the participants were not described).^13^

### Non-Pharmacological Treatment – Potential of Neuromodulation

Transcutaneous vagal nerve stimulation (tVNS) is a method to increase PNS tone, which non-invasively involves electrical stimulation to the parasympathetic vagus nerve and exerts antinociceptive effects. The cervical or auricular branch of the vagus nerve is located directly under the skin, making it a suitable target for tVNS. Recently, there have been several reports showing the analgesic effect of tVNS on pain conditions such as migraine,^124^ rheumatoid arthritis,^112^ fibromyalgia,^113^, and IBS (often comorbid with IBD).^114^ Furthermore, we have demonstrated in healthy subjects that tVNS reduced acid-induced esophageal pain.^202^ These previous reports imply that tVNS has the potential to treat post-inflammatory abdominal pain in IBD.

Although this may be outside of the scope of this review, PNS also has a pivotal role in modulating inflammation through the cholinergic anti-inflammatory pathway.^203^ Our group demonstrated a reduction in TNF-α levels 24 hours after tVNS in healthy subjects.^204^ Moreover, in CD, vagal nerve stimulation by an implanted device reduced the disease activity index and TNF-α level.^205^

Thus, tVNS is expected to be effective for both inflammation and abdominal pain in patients with IBD. To the best of our knowledge, there has been no report demonstrating the effect of tVNS on post-inflammatory pain in patients with IBD thus far. However, it may become a good option for treating post-inflammatory abdominal pain in patients with IBD in the future.

## What are the Unmet Clinical and Research Needs?

The psycho-physiological risk factors and molecular mechanisms of post-inflammatory abdominal pain have been investigated and these appear to influence each other reciprocally.^13^ However, the true extent of this interaction and to what degree each factor contributes to post-inflammatory abdominal pain is not fully understood. Therefore, a comprehensive study investigating the interaction between factors is needed. Ideally, subtypes of post-inflammatory pain would be identified with a combination of detailed psycho-physiological data and multi-omics data (e.g., genomics, transcriptomics, proteomics, lipidomics, metabolomics, etc.), which would be helpful for a better understanding of the mechanism of post-inflammatory abdominal pain. Furthermore, the natural course of post-inflammatory abdominal pain remains unknown due to the lack of prospective studies. Hence, a prospective study with a large sample size would also be needed to understand the nature of post-inflammatory abdominal pain.

In clinical settings of IBD management, post-inflammatory abdominal pain is often inadequately managed. For example, Zeits J. et al. reported that 216 out of 896 IBD patients with pain (24%) received no pain treatment.^5^ Therefore, it is important to increase the awareness of chronic IBD-related abdominal pain to clinicians. Furthermore, as described in another section, there is no guideline available specifically for the treatment of post-inflammatory abdominal pain. Therefore, to achieve optimal outcomes, a robust treatment guideline specific for post-inflammatory abdominal pain is warranted.

To date, the possibilities of new treatments targeting molecular mechanisms or psycho-physiological risk factors have been sought. Although there have been severe side effects or a lack of analgesic effects in pharmacological treatment targeting TRPV1, the preliminary results of new drugs targeting TRPV1 associated mediators seem promising so far. However, those new drugs are still in their infancy, and further studies are needed to determine their effectiveness and safety. What makes it difficult to develop a new drug is that not all drugs effective in animal models of visceral pain have shown efficacy in human clinical trials, suggesting innate differences in the mechanisms of visceral pain between animal models and humans.^206^ Experimental approaches using resected human tissues (e.g., ileum, colon, and rectum flat-sheet preparations and tubular appendiceal preparations) can bridge the gap between the animal models and humans, and can contribute to the development of new effective drugs.^206^

Concerning non-pharmacological treatments, psychological approaches or tVNS seem to have promising potential for analgesic effects without serious side effects. However, clinical studies regarding those therapies have been small and have had limitations in their study design. Therefore, well-organized studies with a large sample size are needed for definitive conclusions.

## Conclusions

This review described the latest knowledge of the prevalence, present clinical practice, possible mechanisms, risk factors, and potential future post-inflammatory abdominal pain treatments. There have been lots of advances in the understanding of the pathophysiology of post-inflammatory abdominal pain in patients with IBD. However, further studies are needed to understand its complex mechanism and nature.

Clinically, post-inflammatory abdominal pain is a widespread symptom in IBD, but it has been often undertreated. Furthermore, medications for pain treatment can exacerbate abdominal pain if they are improperly used. Therefore, a standard treatment strategy based on robust evidence should be established. Furthermore, since the current treatment options are limited and often not effective enough, new treatments in line with its pathophysiology are warranted for optimal outcomes.

## Data Availability

No new data were created or analyzed in this manuscript.
